# Supplementary material for: Drug repurposing for aging research using model organisms
Source: Aging Cell. 2017 Jun 16;16(5):1006–15. doi: 10.1111/acel.12626 (PMC5595691; doi:10.1111/acel.12626)
Supplement: Supplementary file 7 — Data S1 Zip‐Archive of all report cards. [file ACEL-16-1006-s007.zip › RC_ACM.pdf]

ACM

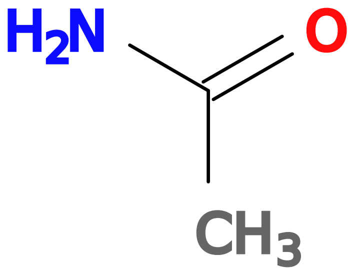

Database identifiers

ChEMBLCompound	CHEMBL16081
DrugBank	DB02736
CHEBI	27856
CHEBI	49028
ZINC	ZINC08034818
eMolecules	27677444
eMolecules	474292

Ranking

	Rank	Score
Drosophila	442/697	0.324
C. elegans	223/591	0.235

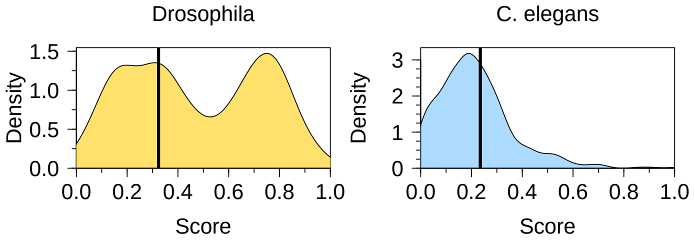

	Ageing implication	Domain conservation	Binding site conservation	Binding affinity	Bioavailability	Lipinski	Promiscuity	Purchasability	Drug approval	**Total**
Drosophila	1.0	0.886	0.719	0.262	(0.9)	0.0	-0.001	0.1	0.075	0.324
C. elegans	1.0	0.933	1.0	0.262	0.25	0.0	-0.001	0.1	0.075	0.235

Names

- **acetimidic acid**
- Acetamide
- ethanimidic acid

Roles

ChEBI entry 49028 has no roles

Status

Approved drug (according to ChEMBL)	No
Classification (according to DrugBank)	experimental
Number of Rule of 5 violations	0
Binding affinity to original target in log units (RF-Score prediction)	3.96
Burns *C. elegans* bioavailability prediction	-2.09

Compound Target Characteristics

Glutathione reductase, mitochondrial

Best gene implication in ageing for this target family came from gene P91938 annotated in UniProt release 2014_02. Annotation GO 8340 (determination of adult lifespan) was Inferred from Mutant Phenotype

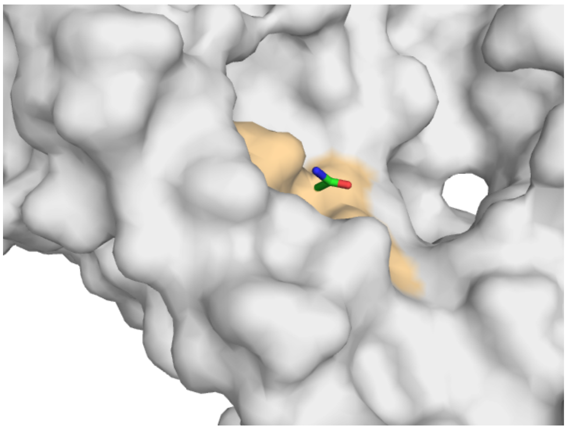

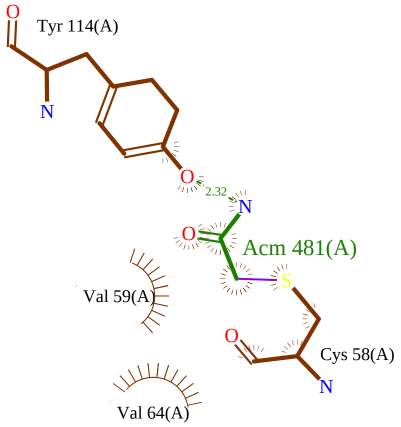

protein	amino acids contacts (binding site)
PDB:1grf:chainA:P00390	C V V Y
tr:H0YC68:H0YC68_HUMAN	C V V Y
sp:P00390:GSHR_HUMAN	C V V Y
tr:F1LRE1:F1LRE1_RAT	- - - Y
sp:P47791:GSHR_MOUSE	C V V Y
sp:P91938:TRXR1_DROME	C V I T
tr:Q86DB5:Q86DB5_CAEEL	C V V Y
tr:Q93379:Q93379_CAEEL	C V V Y
sp:P41921:GSHR_YEAST	C V V Y

	whole protein		domain-based		contact-based	
protein	ident	simil	ident	simil	ident	simil
PDB:1grf:chainA:P00390	1.0	1.0	1.0	1.0	1.0	1.0
tr:H0YC68:H0YC68_HUMAN	0.22	0.23	0.5	0.52	1.0	1.0
sp:P00390:GSHR_HUMAN	1.0	1.0	1.0	1.0	1.0	1.0
tr:F1LRE1:F1LRE1_RAT	0.68	0.76	0.62	0.72	0.25	0.0
sp:P47791:GSHR_MOUSE	0.8	0.89	0.86	0.96	1.0	1.0
sp:P91938:TRXR1_DROME	0.28	0.64	0.36	0.73	0.5	0.72
tr:Q86DB5:Q86DB5_CAEEL	0.51	0.74	0.6	0.86	1.0	1.0
tr:Q93379:Q93379_CAEEL	0.5	0.73	0.6	0.86	1.0	1.0
sp:P41921:GSHR_YEAST	0.44	0.73	0.49	0.77	1.0	1.0

Trxr-1 (FBgn0020653) associated phenotypes

aging defective, developmental rate defective, lethal - all die before end of P-stage, lethal - all die before end of pupal stage, neuroanatomy defective, partially, partially lethal - majority die, some die during pharate adult stage, some die during pupal stage, stress response defective

(Information from FlyBase)

Trxr-1 (UniProt:P91938) annotation

Function: Thioredoxin system is a major player in glutathione metabolism, due to the demonstrated absence of a glutathione reductase. Functionally interacts with the Sod/Cat reactive oxidation species (ROS) defense system and thereby has a role in preadult development and life span. Lack of a glutathione reductase suggests antioxidant defense in Drosophila, and probably in related insects, differs fundamentally from that in other organisms. (PubMed:11158675, PubMed:11525742, PubMed:11796729).

Cofactor: FADNote=Binds 1 FAD per subunit.;

Biophysicochemical properties: Kinetic parameters: KM=6.5 uM for NADPH (at pH 7.4) (PubMed:11158675, PubMed:11782468, PubMed:11796729, PubMed:11877442, PubMed:17385893, PubMed:18211101, PubMed:18991392); KM=1 uM for NADPH (at pH 7.4, 2 mM EDTA, 100 mM KPO(4)) (PubMed:11158675, PubMed:11782468, PubMed:11796729, PubMed:11877442, PubMed:17385893, PubMed:18211101, PubMed:18991392); KM=1 uM for NADPH (isoform B at pH 7.4, 2 mM EDTA, 100 mM KPO(4)) (PubMed:11158675, PubMed:11782468, PubMed:11796729, PubMed:11877442, PubMed:17385893, PubMed:18211101, PubMed:18991392); KM=7.0 uM for dhd (at pH 7.4, 200 uM NADPH, 100 mM KPO(4)) (PubMed:11158675, PubMed:11782468, PubMed:11796729, PubMed:11877442, PubMed:17385893, PubMed:18211101, PubMed:18991392); KM=141 uM for dhd (at pH 7.0, 0.15 mM NADPH, 1 mM EDTA, 1 mg/ml insulin, 50 mM KPO(4), 25 degrees Celsius) (PubMed:11158675, PubMed:11782468, PubMed:11796729, PubMed:11877442, PubMed:17385893, PubMed:18211101, PubMed:18991392); KM=7 uM for dhd (at pH 7.4, 2 mM EDTA, 100 mM KPO(4)) (PubMed:11158675, PubMed:11782468, PubMed:11796729, PubMed:11877442, PubMed:17385893, PubMed:18211101, PubMed:18991392); KM=19 uM for dhd (isoform B at pH 7.4, 2 mM EDTA, 100 mM KPO(4)) (PubMed:11158675, PubMed:11782468, PubMed:11796729, PubMed:11877442, PubMed:17385893, PubMed:18211101, PubMed:18991392); KM=5.9 uM for Trx-2 (at pH 7.4, 100 uM NADPH, 2 mM EDTA, 100 mM KPO(4)) (PubMed:11158675, PubMed:11782468, PubMed:11796729, PubMed:11877442, PubMed:17385893, PubMed:18211101, PubMed:18991392); KM=310 uM for 5,5'-dithiobis-2-nitrobenzoic acid (DTNB) (at pH 7.4, 100 uM NADPH, 100 mM KPO(4)) (PubMed:11158675, PubMed:11782468, PubMed:11796729, PubMed:11877442, PubMed:17385893, PubMed:18211101, PubMed:18991392); KM=0.17 mM for DTNB (at pH 7.0, 0.2 mM NADPH, 10 mM EDTA, 100 mM KPO(4), 25 degrees Celsius) (PubMed:11158675, PubMed:11782468, PubMed:11796729, PubMed:11877442, PubMed:17385893, PubMed:18211101, PubMed:18991392); KM=380 uM for DTNB (at pH 7.4, 2 mM EDTA, 100 mM KPO(4)) (PubMed:11158675, PubMed:11782468, PubMed:11796729, PubMed:11877442, PubMed:17385893, PubMed:18211101, PubMed:18991392); KM=410 uM for DTNB (isoform B at pH 7.4, 2 mM EDTA, 100 mM KPO(4)) (PubMed:11158675, PubMed:11782468, PubMed:11796729, PubMed:11877442, PubMed:17385893, PubMed:18211101, PubMed:18991392); KM=675 uM for methylseleninate (100 uM NADPH) (PubMed:11158675, PubMed:11782468, PubMed:11796729, PubMed:11877442, PubMed:17385893, PubMed:18211101, PubMed:18991392); Vmax=24.3 umol/min/mg enzyme toward NADPH (at pH 7.4) (PubMed:11158675, PubMed:11782468, PubMed:11796729, PubMed:11877442, PubMed:17385893, PubMed:18211101, PubMed:18991392); Vmax=16 umol/min/mg enzyme toward Trx-2 (at pH 7.4, 100 uM NADPH, 2 mM EDTA, 100 mM KPO(4), 25 degrees Celsius) (PubMed:11158675, PubMed:11782468, PubMed:11796729, PubMed:11877442, PubMed:17385893, PubMed:18211101, PubMed:18991392); Note=Measurements were conducted with isoform A unless noted otherwise.; pH dependence: Optimum pH is 7.6 for isoform A with Trx-2 and NADPH as substrates. (PubMed:11158675, PubMed:11782468, PubMed:11796729, PubMed:11877442, PubMed:17385893, PubMed:18211101, PubMed:18991392);

Subunit: Homodimer. (PubMed:17385893).

Subcellular location: Isoform B: Mitochondrion.

Subcellular location: Isoform A: Cytoplasm.

Tissue specificity: During embryogenesis, expression is seen in germ cell progenitors, developing midgut, hindgut and proventriculus. (PubMed:11525742).

Developmental stage: Expressed both maternally and zygotically during all stages of development, highest expression during adult stages. (PubMed:11525742, PubMed:11796729).

Miscellaneous: The active site is a redox-active disulfide bond.
Caution: Was originally thought to be a glutathione reductase. (PubMed:9056265).

(Information from UniProt)

gsr-1 (WBGene00008117) associated phenotypes

lethal, sterile

(Information from WormBase)

Histone acetyltransferase KAT6A

Best gene implication in ageing for this target family came from gene Q92794 annotated in UniProt release 2014_02. Annotation GO subterm of 7568 (aging) was Inferred from Mutant Phenotype

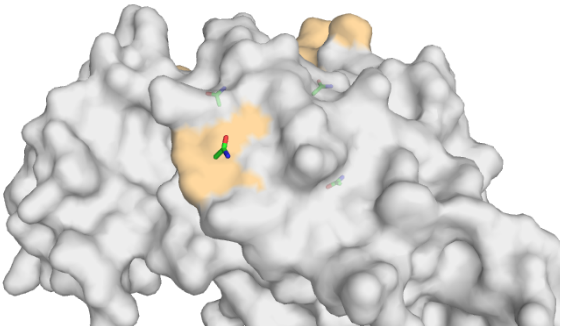

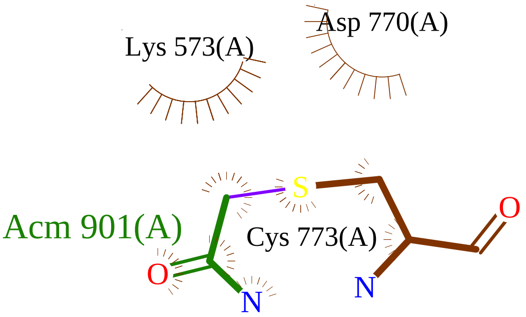

protein	amino acids contacts (binding site)															
PDB:2ozu:chainA:Q92794	K	X	E	H	C	Q	Q	Y	S	C	S	C	P	Q	D	C
tr:C9K0F9:C9K0F9_HUMAN	K	K	E	H	C	Q	Q	Y	S	C	S	C	P	Q	D	C
tr:A5PLL3:A5PLL3_HUMAN	K	K	E	H	C	Q	Q	Y	S	C	S	C	P	Q	D	C
tr:A5PKX7:A5PKX7_HUMAN	K	K	E	H	C	Q	Q	Y	S	C	S	C	P	Q	D	C
sp:Q92794:KAT6A_HUMAN	K	K	E	H	C	Q	Q	Y	S	C	S	C	P	Q	D	C
tr:Q3V1G6:Q3V1G6_MOUSE	K	K	E	H	C	Q	Q	Y	S	C	S	C	P	Q	D	C
tr:Q3UPM9:Q3UPM9_MOUSE	K	K	E	H	C	Q	Q	Y	S	C	S	C	P	Q	D	C
tr:G3X940:G3X940_MOUSE	K	K	E	H	C	Q	Q	Y	S	C	S	C	P	Q	D	C
tr:D0IQI5:D0IQI5_DROME	Q	K	E	H	C	T	Q	Y	S	C	T	A	I	S	E	C
tr:Q9W1A9:Q9W1A9_DROME	Q	K	E	H	C	T	Q	Y	S	C	T	A	I	S	E	C
tr:L8E6K3:L8E6K3_CAEEL	K	K	E	Y	E	P	D	N	S	C	G	N	C	H	D	C
tr:Q21789:Q21789_CAEEL	K	K	E	Y	E	P	D	N	S	C	G	N	C	H	D	C
tr:D7SFM3:D7SFM3_CAEEL	K	K	E	Y	E	P	D	N	S	C	G	N	C	H	D	C
tr:K8ESF3:K8ESF3_CAEEL	K	K	E	Y	E	P	D	N	S	C	G	N	C	H	D	C
tr:G5EGU3:G5EGU3_CAEEL	K	K	E	Y	E	P	D	N	S	C	G	N	C	H	D	C
tr:K8ES81:K8ES81_CAEEL	K	K	E	Y	E	P	D	N	S	C	G	N	C	H	D	C
tr:G5EGP0:G5EGP0_CAEEL	K	K	E	Y	E	P	D	N	S	C	G	N	C	H	D	C
tr:K8F7U3:K8F7U3_CAEEL	K	K	E	Y	E	P	D	N	S	C	G	N	C	H	D	C
tr:G5EBH5:G5EBH5_CAEEL	K	K	E	Y	E	P	D	N	S	C	G	N	C	H	D	C
tr:K8ERR8:K8ERR8_CAEEL	K	K	E	Y	E	P	D	N	S	C	G	N	C	H	D	C
sp:Q08649:ESA1_YEAST	D	K	E	E	S	A	D	Y	A	C	S	T	T	T	D	R

protein	whole protein ident	simil	domain-based ident	simil	contact-based ident	simil
PDB:2ozu:chainA:Q92794	1.0	1.0	0.99	1.0	1.0	0.94
tr:C9K0F9:C9K0F9_HUMAN	0.34	0.35	1.0	1.0	0.94	0.95
tr:A5PLL3:A5PLL3_HUMAN	0.41	0.41	1.0	1.0	0.94	0.95
tr:A5PKX7:A5PKX7_HUMAN	0.57	0.57	1.0	1.0	0.94	0.95
sp:Q92794:KAT6A_HUMAN	1.0	1.0	1.0	1.0	0.94	0.95
tr:Q3V1G6:Q3V1G6_MOUSE	0.52	0.55	0.96	0.98	0.94	0.95
tr:Q3UPM9:Q3UPM9_MOUSE	0.57	0.62	0.96	0.98	0.94	0.95
tr:G3X940:G3X940_MOUSE	0.89	0.96	0.96	0.98	0.94	0.95
tr:D0IQI5:D0IQI5_DROME	0.14	0.29	0.52	0.8	0.5	0.62
tr:Q9W1A9:Q9W1A9_DROME	0.19	0.56	0.52	0.8	0.5	0.62
tr:L8E6K3:L8E6K3_CAEEL	0.07	0.17	0.37	0.61	0.38	0.46
tr:Q21789:Q21789_CAEEL	0.08	0.18	0.4	0.71	0.38	0.46
tr:D7SFM3:D7SFM3_CAEEL	0.12	0.39	0.4	0.71	0.38	0.46
tr:K8ESF3:K8ESF3_CAEEL	0.12	0.39	0.4	0.71	0.38	0.46
tr:G5EGU3:G5EGU3_CAEEL	0.12	0.39	0.4	0.71	0.38	0.46
tr:K8ES81:K8ES81_CAEEL	0.12	0.39	0.4	0.71	0.38	0.46
tr:G5EGP0:G5EGP0_CAEEL	0.11	0.35	0.4	0.71	0.38	0.46
tr:K8F7U3:K8F7U3_CAEEL	0.11	0.35	0.4	0.71	0.38	0.46
tr:G5EBH5:G5EBH5_CAEEL	0.11	0.34	0.4	0.71	0.38	0.46
tr:K8ERR8:K8ERR8_CAEEL	0.11	0.34	0.4	0.71	0.38	0.46
sp:Q08649:ESA1_YEAST	0.08	0.16	0.44	0.71	0.31	0.49

enok-RA (UniProt:D0IQI5) annotation
Subcellular location: Nucleus ECO:0000256—RuleBase:RU361211.

(Information from UniProt)

enok (FBgn0034975) associated phenotypes
female semi-sterile, germline clone, neuroanatomy defective, size defective, somatic clone

(Information from FlyBase)

enok (UniProt:Q9W1A9) annotation
Subcellular location: Nucleus ECO:0000256—RuleBase:RU361211.

(Information from UniProt)

lsy-12 (WBGene00045419) associated phenotypes
egg laying defective, locomotion variant, loss of asymmetry ASE, protruding vulva, reduced brood size, sick

(Information from WormBase)

lsy-12 (UniProt:L8E6K3) annotation
Subcellular location: Nucleus ECO:0000256—RuleBase:RU361211.

(Information from UniProt)

lsy-12 (UniProt:Q21789) annotation
Subcellular location: Nucleus ECO:0000256—RuleBase:RU361211.

(Information from UniProt)

lsy-12 (UniProt:D7SFM3) annotation
Subcellular location: Nucleus ECO:0000256—RuleBase:RU361211.

(Information from UniProt)

lsy-12 (UniProt:K8ESF3) annotation
Subcellular location: Nucleus ECO:0000256—RuleBase:RU361211.

(Information from UniProt)

lsy-12 (UniProt:G5EGU3) annotation
Subcellular location: Nucleus ECO:0000256—RuleBase:RU361211.

(Information from UniProt)

lsy-12 (UniProt:K8ES81) annotation
Subcellular location: Nucleus ECO:0000256—RuleBase:RU361211.

(Information from UniProt)

lsy-12 (UniProt:G5EGP0) annotation
Subcellular location: Nucleus ECO:0000256—RuleBase:RU361211.

(Information from UniProt)

lsy-12 (UniProt:K8F7U3) annotation
Subcellular location: Nucleus ECO:0000256—RuleBase:RU361211.

(Information from UniProt)

lsy-12 (UniProt:G5EBH5) annotation
Subcellular location: Nucleus ECO:0000256—RuleBase:RU361211.

(Information from UniProt)

lsy-12 (UniProt:K8ERR8) annotation
Subcellular location: Nucleus ECO:0000256—RuleBase:RU361211.

(Information from UniProt)
